# Solid Dispersion Approach Improving Dissolution Rate of Stiripentol: a Novel Antiepileptic Drug

**Published:** 2015

**Authors:** Samar Afifi

**Affiliations:** a *Department of Pharmaceutics, College of Pharmacy, King Saud University, Riyadh, Saudi Arabia.*; b *Department of Pharmaceutics, National Organization for Drug Control and Research, Giza, Egypt.*

**Keywords:** Stiripentol, Solid dispersion, PEG-6000, Antiepileptic drugs

## Abstract

Some drugs have low bioavailability due to their poor aqueous solubility and/or slow dissolution rate in biological fluids. Stiripentol (STP) is a novel anticonvulsant drug that is structurally unrelated to the currently available antiepileptics. It has poor aqueous solubility and its solubility has to be enhanced accordingly. Polyethyleneglycol 6000 (PEG-6000) is commonly utilized as a hydrophilic carrier for poorly water soluble drugs in order to improve their bioavailability. STP and PEG-6000 binary system was obtained by physical mixture, solvent evaporation, co-evaporation and melting methods using different weight ratios. The properties of the prepared binary systems were evaluated using dissolution rate, phase solubility, Fourier-transform infrared (FTIR) spectroscopy, differential scanning calorimetry (DSC) and scanning electron microscope (SEM) studies. The FTIR spectroscopic studies showed the stability of STP and absence of STP-PEG-6000 interaction. The DSC and SEM studies indicated the amorphous state of STP in its binary systems with PEG-6000.

Dissolution profile of STP was significantly improved *via* complexation with PEG-6000 as compared with the pure drug. The binary system which was prepared using melting method showed the highest dissolution rate. The promising results of the prepared binary systems open the avenue for further oral formulation of STP.

## Introduction

Oral bioavailability of a drug is based on its solubility and/or dissolution rate. Dissolution may be the rate determining step for a medical effect to appear. Accordingly, efforts to increase dissolution of drug with limited water solubility is a valuable target. The oral formulation of poorly water-soluble drugs is one of the major problems in pharmaceutical manufacturing. Many approaches are available to solve this problem, including salt formation, micronization and addition of solvent or surface active agents. Solid dispersion (SD) is one of these approaches which involves a dispersion of one or more active ingredients in an inner carrier or matrix in solid state prepared by solvent evaporation or melting method (1).

**Figure 1:**
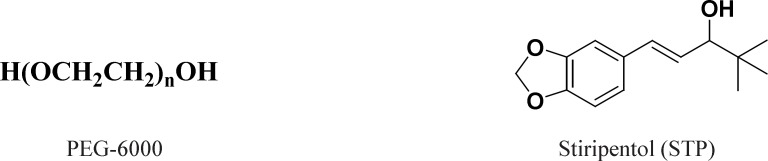
The chemical structure of Stiripentol and PEG-6000.

Polyethylene glycols (PEGs) with molecular weights of 1, 500-20,000 are among the several carriers which have been employed in preparing solid dispersions. They are widely used due to their low melting point, low toxicity, wide drug compatibility and hydrophilicity (2). Solubility of PEGs in water is generally good, but it decreases with increase in their molecular weight. Solubility of PEGs in many organic solvents is a particular advantage which enables their use in SDs formation (3). The relatively low melting points of PEGs are advantageous for manufacturing of SDs using melting method. Furthermore, PEGs have the ability to solubilize some poor water soluble compounds and to improve their wettability (4). The SDs of drugs with PEG 6000 (Figure 1) may be useful to solve various problems such as stability, solubility, dissolution and bioavailability (5-7).

Stiripentol (STP) is a novel anticonvulsant drug for the treatment of at least some forms of epilepsy. It is structurally unrelated to any other currently available antiepileptic drugs (AED) (8). STP has good safety profile with relatively high therapeutic index and it is generally well tolerated, even in epileptic children (8). Chemically, STP is a 4, 4-dimethyl-1-[3,4 (methylenedioxy)-phenyl]-1-penten-3-ol (Figure 1).

STP may increase γ-aminobutyric acid (GABA) levels in brain tissues. STP-induced increase in GABA concentration involves at least two independent neurochemical mechanism: inhibition of synaptosomal uptake of GABA and inhibition of GABA transaminase (9, 10). Antieileptic potency of STP has also been proven in different types of seizures in humans (11). Stiripentol is easily soluble in acetone and alcohol, moderately soluble in chloroform and insoluble in water and it is stable in frozen state (12). STP’s bioavailability is relatively low due to its poor water solubility and possible hepatic first-pass and it is highly bound to plasma proteins (13). Additionally, STP is slowly distributed with a characteristic pattern of a multiphasic elimination curve (14, 15).

Literature survey revealed that no attempts have been made to increase the solubility of STP. Therefore, the present study is aimed to formulate different binary systems of STP with PEG-6000 in order to improve its aqueous solubility, wettability, *in-vitro* dissolution and hence its bioavailability of the drug. The solid binary systems were prepared taking different drug/polymer ratios using different techniques, namely physical mixture, solvent evaporation, co-evaporation and melting techniques. After assessing the drug content in the binary systems, the products were characterized by differential scanning calorimetry (DSC), fourier transformation infrared spectroscopy (FTIR) and scanning electron microscope (SEM). Moreover, solubility and dissolution rate studies were performed to evaluate the prepared binary systems of STP with PEG-6000 as compared with that of STP alone.

## Experimental

*Materials*

Stiripentol (STP) was obtained from Sigma Chemical Co. (St. Louis, MO, USA). Polyethylene glycol 6000 (PEG-6000) was purchased from S.D. Fine Chemicals (Mumbai, India). Other chemicals and reagents were of analytical grade. Distilled water was used throughout the studies.

*Methods*

*Preparation of STP binary systems*

To obtain physical mixtures (PMs). The required amounts of drug and carrier were geometrically mixed (previously screened through 100-mesh) for 20 min in a glass mortar with the help of stainless steel spatula. PMs were prepared for STP: PEG-6000 ratios 1:1 and 1:2 and were coded PM-1 and PM-2, respectively. The binary systems were also prepared by solvent evaporation method according to the compositions presented in Table 1. The PEG-6000 was dissolved in ethanol under stirring, STP (100 mg) was then added and stirring was continued for 45 min. The organic solvent was removed at ambient temperature under vacuum till constant weight was achieved. The resultant dried solid systems were stored in a desiccator at room temperature for 24 h before pulverization and sieving through 100-mesh (16). The obtained binary systems were coded as SE-1 and SE-2 (Table 1).

The binary systems of STP (100 mg) with PEG-6000 were also prepared using melting method in 1: 1 and 1: 2 weight ratios. PEG-6000 was melted at 50-60ºC in preheated dish on water bath. The STP (100 mg) powder was added to the melted PEG-6000 with continuous stirring. The mass obtained was cooled to room temperature (7, 17). The prepared solid systems were stored in a desiccator for 24 h then crushed utilizing porcelain mortar pestle and sieved through 100-mesh. The binary systems were named as MM-1 and MM-2 (Table 1). Additionally, the binary systems of STP and PEG-6000 were performed using co-evaporated method (CE). An aqueous solution of PEG-6000 was added to alcoholic solution of STP. The resulting mixtures were dried using magnetic stirrer which was maintained at 40-50ºC by continuous stirring for 6 h. The dried mass was pulverized and sieved through 100-mesh (18). The prepared binary systems were named as CE-1 and CE-2 (Table 1). 

**Table 1:** The composition of the prepared binary systems of STP/PEG-6000

Formulation code	Drug: polymer ratio	Method used
PM-1	1:1	Physical mixture
PM-2	1:2	Physical mixture
SE-1	1:1	Solvent evaporation
SE-2	1:2	Solvent evaporation
MM-1	1:1	Melting method
MM-2	1:2	Melting method
CE-1	1:1	Co-evaporation
CE-2	1:2	Co-evaporation

*Percentage yield*

The practical percentage yields were calculated to determine the efficiency of the methods which were used for the preparation of binary systems and it helps in selection of appropriate method of production. Solid mixtures were collected and weighed to determine practical yield (PY) from the following equation:

Equation (1) Percentage yield=Practical weight of Solid mixture Theoretical weight of Solid mixture×100

*Drug content*

Binary systems equivalent to 10 mg of STP were weighed accurately and dissolved in suitable quantity of methanol (10 mL). This solution was vortexed for one minute. The solution was filtered through filter paper, and diluted suitably. Drug content was analyzed at 301 nm by UV spectrophotometer (Shimadzu UV3300PC, Tokyo, Japan). Drug content was determined from the standard plot. Each sample analyzed in triplicate (17, 19). 

*Characterization of binary systems*

*Differential scanning calorimetry (DSC)*

The powdered sample (3-5 mg) was hermetically sealed in aluminum pans and heated at a constant rate of 10°C/min, over a temperature range of 25-200°C. Thermograms of the samples were obtained using differential scanning calorimetry (DSC-60, Shimadzu, Japan). Thermal analysis data were recorded using a TA 50I PC system with Shimadzu software programs. Indium standard was used to calibrate the DSC temperature and enthalpy scale. N_2_was used as purging gas at rate of 30 mL/min (20).

*Fourier transform infrared spectroscopy (FTIR)*

The FTIR spectra for STP binary systems were performed using Perkin-Elmer FTIR series (model-1615) spectrophotometer. Samples were mixed with potassium bromide (KBr) and compressed into discs. The scanning range was between 4000-450 cm^-1 ^(21).

*Scanning electron microscopy study (SEM)*

The surface morphology of STP and its binary systems were examined under scanning electron microscope (Jeol, JSM-6360 LV scanning microscope, Tokyo, Japan). Before microscopy, the dried samples were mounted at carbon tape and were sputter-coated using gold (Jeol, JFC-1100 fine coat ion sputter, Tokyo, Japan). The photomicrographies were taken at an excitation voltage of 20 kV. The selected magnification was 1000 × since it was enough to appreciate the general morphology of the powders under study (20).

*Solubility determination*

The effect of different concentrations of PEG-6000 on the equilibrium solubility of STP in distilled water at 37ºC was studied by adding an excess of STP (20 mg) to distilled water (20 ml) and varying concentrations of PEG-6000. The samples were placed in a shaking water bath (Julabo SW22, Seelbach, Germany), agitated at 50 rpm for 48 h. Then aliquots were filtered through a Millipore filter (0.45 µm) and the filtered samples were diluted suitably and assayed spectrophotometrically at 301 nm, a wavelength at which PEG-6000 does not interfere. Three determinations were carried out for each sample to calculate the solubility of STP (22).

*Dissolution rate studies*

All the prepared formulations were subjected to *in-vitro* dissolution tests in the media of 0.1N HCl (900 mL) with 0.25% w/v sodium lauryl sulphate (SLS) for a period of 90 min using USP-II apparatus (Erweka DT 600, Heusenstamm, Germany). The prepared binary systems equivalent to 100 mg STP were weighed and dispersed on the media maintained at 37 ± 0.5˚C at speed of 50 rpm; 5 milliliter of samples were withdrawn at regular time intervals of 10, 20, 30, 45, 60, and 90 min with a syringe filter (0.45 µm) (16, 23). The volume of dissolution fluid was adjusted to 900 ml by replacing each 5 mL aliquot withdrawn with 5 mL fresh dissolution media (24, 25). The filtered samples were suitably diluted. The concentration of STP in each sample was determined by UV spectrophotometer at 301 nm and the data were analyzed by standard curve equation. Under the experiment conditions, PEG-6000 did not interfere with spectrophotometric assay. The mean of at least three determinations was used to calculate the drug release.

*Release kinetics*

To study the release kinetics, the data obtained from *in-vitro* drug release studies were analyzed using various kinetic models.

The zero order rate describes concentration independent drug release rate from the formulation (Equation 2). 

$C=K_{0t}$

 Equation (2)

where, K_0_ is zero-order rate constant expressed in units of concentration/time and t is the time.

The first order describes the release from system where release rate is concentration dependent drug release from the system (Equation 3).

$\mathrm{Log C}=\mathrm{Log}C_{0}-\mathrm{Kt}/2.303$

Equation (3)

where, C_0_ is the initial concentration of drug and K is first order constant.

Higuchi̕ model described the release of drug based on Fickian diffusion as a square root of time dependent process from swellable insoluble matrix (Equation 4).

 Equation (4) $$Q={\mathrm{Qt}}^{1/2}$$

where, K is the constant reflecting the design variables of the system. 

The mechanism of the drug release was assessed using Korsmeyer-peppas model derived a simple relationship which described drug release from a polymeric system (Equation 5). To find out the mechanism of drug release, first 60% drug release data was fitted in Korsmeyer–Peppas model.

$\frac{\mathrm{Mt}}{\mathrm{M\infty}}=Kt^{n}$

 Equation (5)

where Mt / M∞ is fraction of drug released at time t, K is the release rate constant incorporating structural and geometric characteristics of the tablet, and n is the release exponent. The n value is used to characterize different release mechanisms (26).

A plot of log cumulative % drug release vs. log time was made. Slope of the line was n. The n value is used to characterize different release mechanisms as given in Table 3, for the cylindrical shaped matrices. Case-II generally refers to the erosion of the polymeric chain and anomalous transport. (Non-Fickian) refers to a combination of both diffusion and erosion controlled-drug release.

*Statistical analysis*

All studies were performed in triplicate and values were expressed as mean ± SD. The data were analyzed by two-way analysis of variance (ANOVA) and a value of p < 0.05 was considered as significant.

## Results and Discussion

*Yield and drug content*

**Figure 2:**
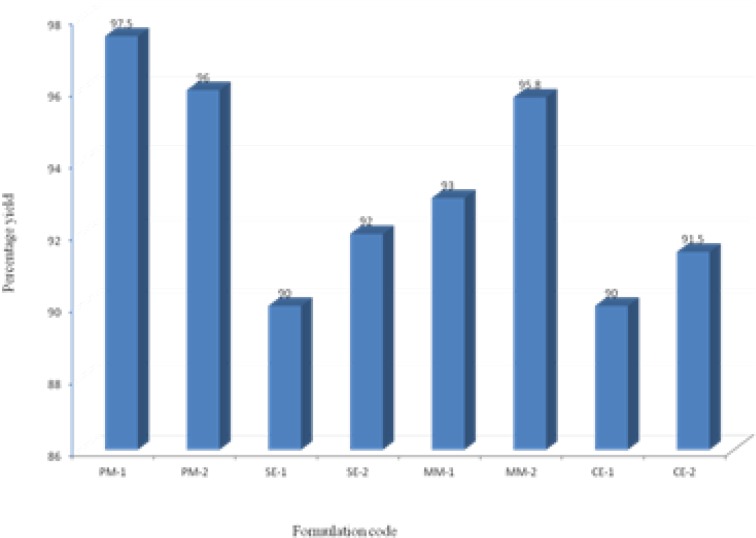
Percentage yield of all formulations

The prepared binary systems were collected and were found to be free flowing and off-white in color. Practical yields were calculated for all formulations (Figure 2). All formulations showed good yields in the range of 90-97.5%. Binary system of physical mixtures and melting method gave better yields than that of solvent evaporation and co-evaporation methods.

The content of STP in each preparation was assayed by UV spectroscopy. The assay values were between 98.5% and 101% of the theoretical values indicating the applications of the present method for the preparation of binary systems with high content uniformity.

*DSC studies*

The thermograms of the pure drug (STP), the carrier (PEG 6000), PMs, SEs, MMs and CE are illustrated in Figures 3a and 3b. The DSC thermograms of each component exhibited a sharp endothermal peak corresponding to the melting point of STP (82°C) and PEG 6000 (61.58°C). Thermal traces for PM-1 showed a weak broad two peaks shifted to lower melting point. In case of PM-2 the intensity of the drug peak was decreased than in PM-1 which may be due to decrease the crystallinity of the drug. While the slight difference in heat of fusion of PM indicates a slight reduction in PEG crystallinity. The thermal profiles of SE-1 and SE-2 exhibited a single endothermic peak at 52°C corresponding to the fusion of the carrier while no peaks were detected representing the melting of the drug. MM-1 and MM-2 have single endothermic peak at 48°C showing that the solid drug was dissolved completely into the molten PEG 6000 and no more drug was present in its undissolved form inside the system. Complete disappearance of the drug peak in SEs, MMs and CEs demonstrated that the drug was converted from crystalline to amorphous form or the drug was dissolved in the melted carrier before reaching its fusion temperature. This phenomenon is already observed in solid dispersions of other drugs with PEG(25). STP-PEG 6000 systems were found to be completely miscible in the liquid phases and completely immiscible in the solid state.

**Figure 3a:**
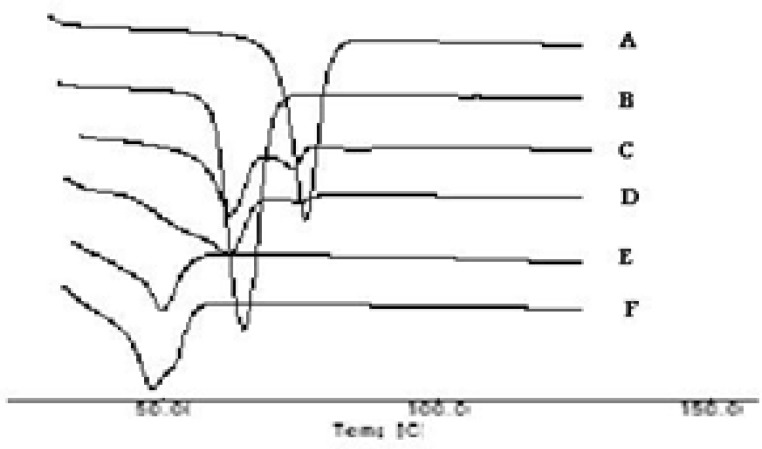
DSC Thermographs of Solid systems: STP (A), PEG 6000 (B), PM-1 (C), PM-2 (D), SE-1 (E) and SE-2 (F).

**Figure 3b:**
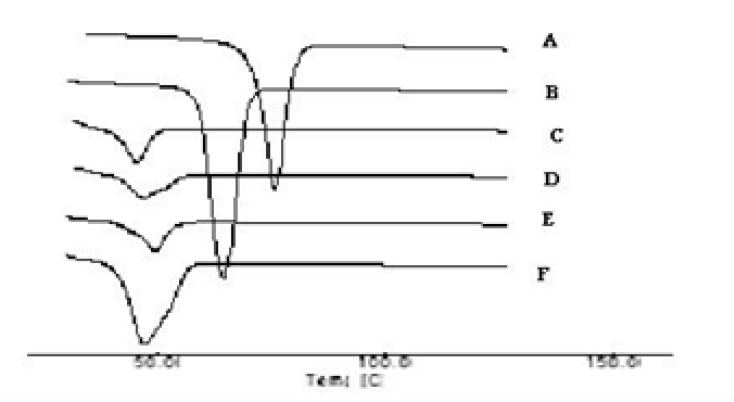
DSC Thermographs of Solid systems: STP (A), PEG 6000 (B), MM-1 (C), MM-2 (D), CE-1 (E) and CE-2 (F).

*Fourier Transformation Infrared Spectroscopy (FTIR)*

FTIR spectroscopic studies were conducted to detect possible drug: carrier interaction. The complexes were compared with both pure STP pure PEG-6000.

**Figure 4:**
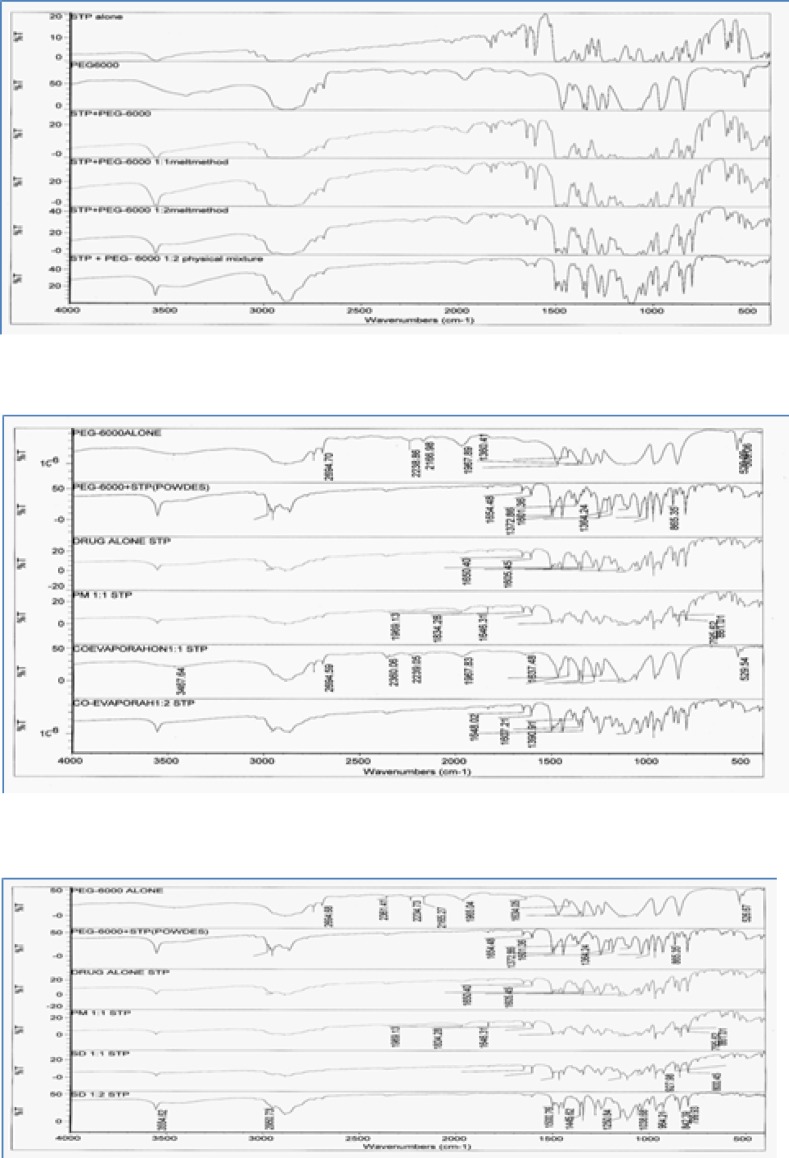
FTIR spectra of Stiripentol and all its binary systems with hydrophilic polymerPEG-6000.

IR spectra of the prepared binary systems (Figure 4) incorporating STP and PEG-6000 in different ratios showed IR peaks of STP and the carrier and no any additional peaks were observed indicating no significant chemical interaction has been occurred between STP and the carrier. IR spectrum of STP was characterized by a sharp peak at 3554.67 cm^−1^ corresponding to 2° OH group and broad band at 2953.97 – 2890.02 cm^−1^ indicating HC = CH and aliphatic CH. When the drug was incorporated with PEG-6000 in different ratios using several methods their respective peaks are not distributed in the observed IR concluding that there was no drug polymer interaction. No additional peak was observed in all binary systems indicating absence of any chemical interaction between STP and the polymer. That confirms the stability of the drug with its binary systems.

*Scanning electron microscopy*

**Figure 5:**
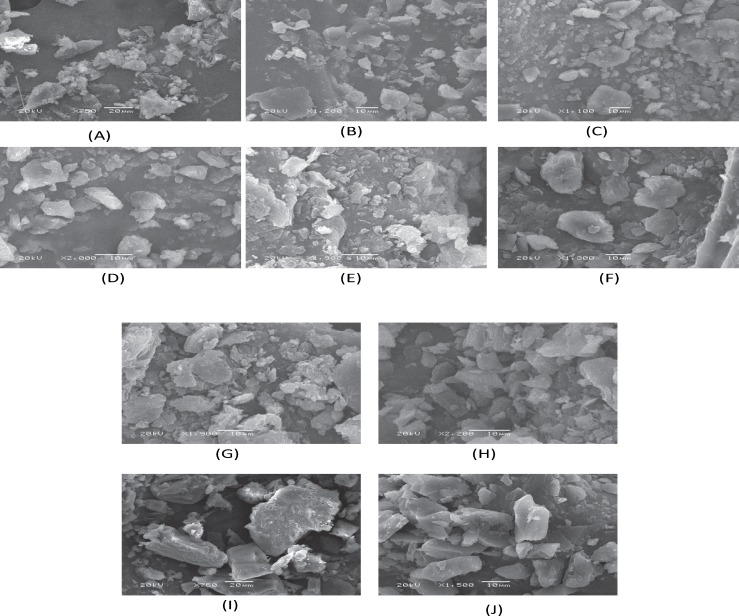
SEM images of (A) Stiripentol (B) PEG-6000 (C) PM-1 (D) PM-2 (E) SE-1 (F) SE-2 (G) MM-1 (H) MM-2 (I) CE-1 (J) CE-2.

The surface morphology of the STP and its binary systems was examined by SEM analysis. Figure 5 shows some selected SEM images of representative samples. 

The results of SEM studies were in agreement with DSC thermograms. It was not possible to distinguish pure components in case of binary systems prepared by melting, solid evaporation and co-evaporation methods indicating uniform distribution of STP in the polymer matrix. However, the corresponding physical mixtures were appeared as the combination of characteristics of pure STP and the carrier. The STP crystals appeared as fine particles with smooth surfaces partially agglomerated in bundles. The PEG-6000 exhibited crystalline agglomerates of rather irregular size and shape, which are clearly visible in PMs. 

The presence of less crystalline STP, uniformly and finely dispersed or adhered to the carrier surface was observed in the SEs, MMs, and CEs. The drug and the polymer are in the state of solid mass where the majority of the drug particles were observed to be dissolved in the polymer. 

These observations provide the evidence of solid mass formation and are in accordance to the results obtained from FTIR and DSC. 

*Phase solubility study*

**Figure 6:**
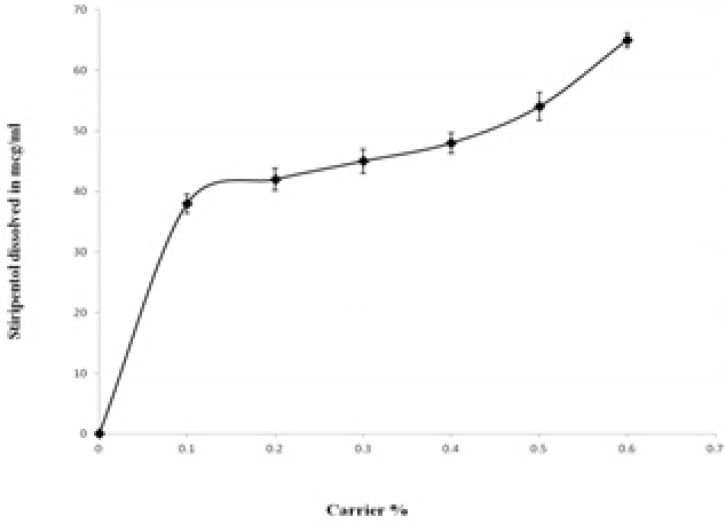
Phase solubility diagram for Stiripentol and PEG 6000 (Carrier).

The solubility of STP in distilled water was found to be 6.03 µg/mL. The influence of the PEG- 6000 upon the solubility of STP is presented in Figure 6. The solubility of STP was increased with respect to weight fraction of the carrier. At 0.6% of PEG-6000 the increase in the solubility was ~ 10 fold compared with the pure drug. The increase in the solubility with increase in PEG-6000 concentrations indicates the solvent properties of PEG-6000 for the STP which is in agreement with several previous studies on other poorly soluble drugs (22, 25). The increase in the solubility in the presence of PEG-6000 can probably be explained by increased wettability of STP. Indeed, PEG-6000 causes a decrease of the interfacial tension between the drug and the dissolution medium. The PEG-6000 is a hydrophilic polymer, therefore, it confers more hydrophilicity upon the hydrophobic STP particles, enhancing wettability of drug particles and consequently increases the particles dissolution. The hydroxyl groups of the polymer can interact *via* H-bonding with both hydroxyl and benzodioxole moieties of STP in solution. Additionally, the plot of drug solubility against polymer concentration at 37°C indicated a linear relationship between drug and polymer solution.

*Dissolution studies*

The *in-vitro* release study of poorly water-soluble drugs requires a dissolution medium entirely different from those used for water soluble drugs. The incorporation of a small amount of surfactant in the dissolution medium is one of the useful techniques in dissolution of insoluble drugs (27). Utilizing surfactant in the dissolution medium may be physiologically meaningful, due to the presence of natural surfactants (as bile salts) in the gastrointestinal tract. Surfactants are able to accelerate the *in-vitro* dissolution of poorly water-soluble drugs due to wetting, micellar solubilization, and /or deflocculation. Studies on sodium lauryl sulphate, as a surfactant, have shown to satisfy these needs (28). Accordingly, in the present study dissolution of pure STP, PMs, SEs, MMs, CEs were carried out in 0.1N HCl containing 0.25 % w/v sodium lauryl sulphate.

**Figure 7:**
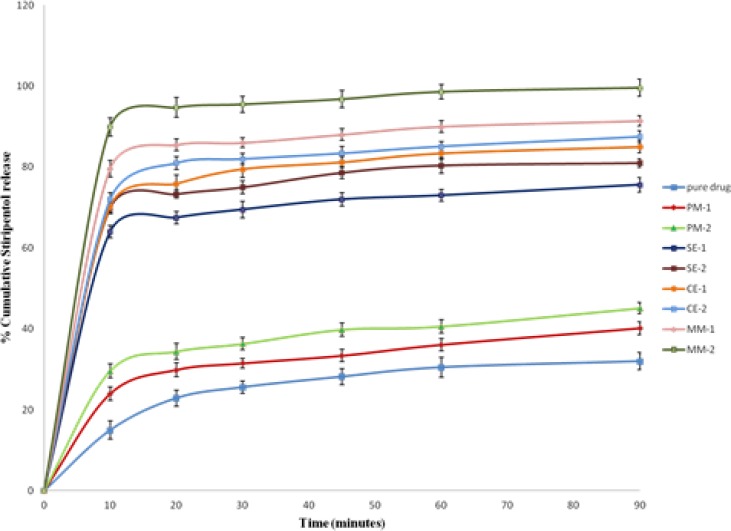
*In-vitro* dissolution profile of Stiripentol and its binary systems of Stiripentol with PEG 6000 in pH 1.2 buffers.

*In-vitro* dissolution profile for STP and its binary systems are shown in Figure 7. *In-vitro* drug release data revealed that all STP binary systems showed significant increase in the dissolution rate of STP as compared with pure drug (p < 0.05).PEG-6000 physical mixtures showed a slight, but significant enhancement in dissolution efficiency (p < 0.05). Dry mixing of the drug with a hydrophilic carrier in PMs results in greater wetting and increase surface available for dissolution by reducing the interfacial tension between the hydrophobic drug and dissolution media (29). It was also possibly due to the close contact of the drug with the hydrophilic polymer, brought about by the dry mixing process (30).

The increase in dissolution rate of binary system prepared by melting method was more significant than the increase in dissolution rate of binary systems prepared by solvent evaporation and co-evaporation methods as shown in Figure 7 (p < 0.05). This can be attributed to the uniform and homogenous distribution of the drug in the polymer crust in a highly dispersed state as a result of melt in procedure. Complete homogenous inclusion of the drug particles in the polymer matrix was achieved by incorporating the drug in the melted PEG-6000 with gentle mixing using a porcelain pestle. Thus, when a binary system comes in contact with an aqueous dissolution medium, the hydrophilic carrier dissolves and results in precipitation of the embedded drug into fine particles, which increase the available dissolution surface (31). Melting method allowed better incorporation of the drug in the carrier than the solvent evaporation and co-evaporation.

The entrapment of the drug molecules in the polymer matrix during solvent evaporation may improve the wetting of drug particles and localized solubilization by the hydrophilic polymeric carriers. Also due to presence of drug in amorphous state as compared the physical mixtures and pure drug, where drug is present in crystalline state (32).

Drug dissolution rates were comparably high for compacted granules then their physical mixture and drug alone, suggesting that compaction processes with hydrophilic polymers improve the drug dissolution rate by keeping the polymer and the drug particles in close proximity during dissolution (33).

The dissolution behavior of pure STP and its binary systems with PEG-6000 in various ratios have been explored in terms of dissolution efficiency at 10 min (DE_10_) as well as percent drug dissolved at 10 and 30 min (DP_10_ and DP_30_) and the relative dissolution rate (RDR) at 10 min in comparison with the pure drug. The dissolution rates of pure drug and its physical mixtures were found to be 32, 40.2 and 45.1% for pure drug, PM-1 and PM-2 in 90 min, respectively (Table 2).Whereas in solid dispersions, SE-1 and SE-2 displayed 75.6 and 81% drug release, respectively (Table 2). The binary systems which were prepared by co-evaporation method, CE-1 and CE-2 gave 85 and 87.5% drug release, respectively. The mixtures prepared by melting method using 1:1 and 1:2 ratio showed a pronounced drug release of 91.4 and 99.6% in 90 min, respectively.DP_10_ and DP_30_ values in Table 2 indicated that the onset of dissolution of pure STP is very low as about 25.6% of the drug being dissolved within 30 min. Binary systems with PEG-6000 considerably enhanced dissolution rates within 30 min as compared with pure STP. The value of DE_10_for pure STP (7.5%) was enhanced in PMs to be 14.8, 34.95, 36 and 44.95% in case of SEs, CEs and MMs, respectively. The time needed of the pure STP for 30% release was 60 min which was reduced into 5 min for MM at 1:2 (STP/PEG-6000) ratios.

*In-vitro* release studies revealed that there is a marked increase in the dissolution rate of STP in the all prepared mixtures when compared with pure STP. The increase in dissolution rate was in the following decreasing order: MMs > CEs > SEs > PMs.

The dissolution rate of STP in the binary systems was strongly dependent on the relative concentration of the carrier. As the concentration of the carrier in the mixture increased, the dissolution rate also increased. This may be attributed to the increase in the wettability, conversion to the amorphous form and solubilization of the drug due to hydrophilic carrier and solubilizing effect of surfactant. Generally, dissolution may be described by two processes such as the rate associated with diffusion or transport process of the solvated molecule to bulk part of the dissolution medium and the rate of the interfacial or solid-solvent reaction leading to solubilization of the molecule (34). In the present dissolution study, the increase in dissolution rate of the STP binary systems might be attributed to the formation of hydrogen bonding between polar OH groups in STP and the dissolution media accordingly the wettability increased. While the increase in the dissolution and dissolution efficiency values could be due to the reduction of the interfacial tension between the hydrophobic drug particles and the dissolution medium, owing to the presence of the hydrophilic polymer and a local solubilizing effect acting during early stages of the dissolution process in the microenvironment surrounding the drug particles. The observed increase in dissolution of STP with increase in PEG concentration might be attributed to the increase in the amorphonising efficiency of PEG at higher concentrations (35). A poorly water-soluble drug with strong hydrophobicity results in floating of the drug on the surface of dissolution medium. Therefore, the better wettability and dispensability of a drug in the solid binary systems, will increase the possibility of achieving an increase in drug dissolution (36). During the present dissolution studies, it was noted that drug- carrier binary systems sink immediately, whereas pure drug keeps floating on the surface for a longer time interval. Moreover, PEG may form a concentrated diffusion layer into which the drug dissolves prior to its release into the aqueous medium (25). From the obtained dissolution parameters (Table 2), it was clear that binary systems prepared by melting method were superior to those prepared by solvent evaporation and co-evaporation methods regarding drug dissolution efficiencies. This can be attributed to the uniform and homogenous distribution of the drug in the polymer crust in a highly dispersed state as a result of melt in procedure. Thus, when a binary system comes in contact with an aqueous dissolution medium, the hydrophilic carrier dissolves and results in precipitation of the embedded drug into fine particles, which increase the available dissolution surface (31). Additionally, dissolution rate studies of STP in phosphate buffer (pH 6.8) gave nearly the same results as that in 0.1N HCl. The results revealed that the dissolution rate of STP in its binary systems was dependent on the relative concentrations of the PEG-6000. Moreover, improvement of the solubility and the dissolution rate of STP in its binary systems, which can be formulated as tablets or capsules with better dissolution characteristics. The rapid dissolution of STP from its binary systems may be attributed to the decrease in the drug crystallinity and its molecular and colloidal dispersion in the hydrophilic carrier matrix. As the soluble carrier dissolves, the insoluble drug gets exposed to dissolution medium in the form of very fine particles for quick dissolution (37).

**Table 2:** Dissolution parameters of pure drug and binary systems

Binary systems	aDP_10_ (%)	bDE_10_ (%)	aDP_30_ (%)	RDR_10_	ct_30 _(min)
pure drug STP	15	7.5	25.6	1	60
PM-1	24	12	31.5	1.6	20
PM-2	29.6	14.8	36.3	1.97	15
SE-1	64	32	69.5	4.3	8.6
SE-2	69.9	34.95	75	4.66	8.2
CE-1	70.3	35.15	79.5	4.69	7
CE-2	72	36	82	4.73	6.9
MM-1	79.6	39.8	86	5.31	6.4
MM-2	89.9	44.95	95.5	5.84	5

a DP: dissolution percent,

b DE: dissolution efficiency,

c t_30%_: time to release 30% of the drug

*Kinetic study*

Drug release data were analyzed according to zero order, first order and Higuchi models. Table 3 shows the regression parameters obtained after fitting various release kinetic models to the *in-vitro* dissolution data. The obtained regression coefficients (*R*^2^) for zero-order kinetics, first-order kinetics and Higuchi model were 0.618-0.810, 0.702-0.964 and 0.945-0.995, respectively. The (*R*^2^) values of Higuchi release model were found to be higher than that of zero and first order kinetics. Accordingly, the release of the drug from the prepared binary systems followed predominantly Higuchi model. According to Korsemayer-peppas model n-value was used to characterize different release mechanisms as given in Table 3. The n value was 0.89 which refers that the *in-vitro* release exhibits case II Non-Fickian diffusion refers a combination of both diffusion and erosion release.

**Table 3:** Statistical parameters of various Formulations after fitting drug release data to various release kinetics Models.

Formulations	Zero-order model	First order model	Higuchi model	Release mechanism (Korsemayer-peppas)
	*R* ^2 ^ *K*	*R* ^2 ^ * K *	*R* ^2^ * K*	*R* ^2 ^n
Drug	0.850 1.302	0.813 -0.187	0.923 0.134	0.959 0.887
PM-1	0.810 1.770	0.851 -0.189	0.945 0.138	0.961 0.895
PM-2	0.782 1.350	0.814 -0.167	0.946 0.139	0.970 0.924
SE-1	0.619 0.72	0.702 -0.525	0.995 0.598	0.917 0.792
SE-2	0.629 0.662	0.936 -0.900	0.969 0.507	0.918 0.778
CE-1	0.627 0.633	0.813 -0.82	0.980 0.461	0.917 0.767
CE-2	0.636 0.614	0.964 -0.730	0.965 0.383	0.919 0.761
MM-1	0.639 0.614	0.773 -0.740	0.970 0.396	0.919 0.761
MM-2	0.618 0.541	0.807 -0.400	0.981 0.430	0.918 0.743

## Conclusion

Stiripentol (STP) is practically insoluble in water and aqueous fluids. The present study demonstrated the preparation of STP binary systems with polyethylene glycol 6000 using physical mixture, solvent evaporation, melting and co-evaporation methods. The solubility and dissolution rate properties of STP were improved from its binary systems and to some extent in PMs. The solubility, DSC, FTIR, and SEM studies clarified the physical state of both the drug and the carrier in the samples. Furthermore, No well defined chemical interaction between STP and PEG-6000 in the binary systems was observed. The higher dissolution rates exhibited by the prepared binary systems may imply enhanced oral bioavailability due to increased wetting properties and solubility of drug in the hydrophilic polymer. The prepared STP binary systems open the avenue for further oral formulations of STP.
